# Iron Deficiency Anemia in Teenage Girls: The Impact of Menarche and Nutritional Care

**DOI:** 10.7759/cureus.84997

**Published:** 2025-05-28

**Authors:** Sidra Anwar, Muhammad Kamran Rauf, Mehrin Farooq, Mahnoor Khan, Wajeeha Maqsood, Shaina Gulraiz

**Affiliations:** 1 Medicine, Fatima Memorial Hospital College of Medicine and Dentistry, Lahore, PAK; 2 Medicine, Ghurki Trust Teaching Hospital, Lahore Medical and Dental College, Lahore, PAK; 3 Emergency Medicine, Royal Bournemouth Hospital, Bournemouth, GBR

**Keywords:** age of menarche, dietary counseling, high prevalence, iron-rich food, lack of awareness, menstrual irregularities

## Abstract

Background and aim

Anemia is a condition characterized by a deficiency in red blood cells or hemoglobin (Hb), which significantly impacts adolescent girls, often due to dietary deficiencies and lack of counseling at menarche. This research aims to find the prevalence of iron deficiency anemia (IDA) among teenage girls after menarche, and attending outpatient departments (OPDs) caused by reasons unrelated to pathological anemia.

Materials and methods

This cross-sectional study, conducted at a tertiary care hospital from April 2024 to November 2024, included 165 teenage girls aged 13-19 years with anemia (Hb ≤12), excluding known cases of hemolytic anemia or autoimmune disorders, recent blood loss, significant organ dysfunction, psychiatric illness, and ongoing anemia treatment. Complete blood count (CBC), serum iron, serum ferritin, and total iron-binding capacity (TIBC) were used to assess anemia.

Results

The age of menarche ranged from nine to 16 years, with a mean of 12.5±2.53 years. Fifty-five (33.33%) of participants had moderate anemia (Hb 8-10 g/dL) or severe anemia (Hb<8 g/dL), while 110 (66.67%) either had no anemia or mild anemia (Hb >10 g/dL). One hundred thirty-five (81.8%) were unaware of anemia prevention methods. Significant associations were found between anemia and dietary habits, heavy or irregular menstrual bleeding, supplement use, and symptoms of anemia (fatigue, dizziness, and weakness).

Conclusion

This study showed a high prevalence of IDA in teenage girls, particularly at menarche when iron needs increase. Vast educational plans and awareness campaigns are recommended to be implemented in society for the prevention, early diagnosis, and management of anemia in teenage girls.

## Introduction

Anemia is characterized by a deficiency in red blood cells (RBCs) or hemoglobin (Hb). It remains a prevalent global health issue for various age groups. However, little is known about its prevalence among teenage girls who seek medical care for reasons unrelated to pathological anemia.

The World Health Organization (WHO) defines anemia as a condition in which the quantity of RBCs or their ability to carry oxygen is inadequate to meet physiological demands. Men over the age of 15 (Hb under 130 g/L), women over the age of 15 who are not pregnant (Hb under 120 g/L), and children between the ages of 12 and 14 (Hb under 120 g/L) are considered anemic. Pregnancy requires modifications to anemia definitions because of changes in homeostasis. Anemia during pregnancy is defined as follows: Hb below 110 g/L in the first trimester, 105 g/L in the second and third trimesters, and 100 g/L in the postpartum period. Anemia is categorized using mean corpuscular volume (MCV) as follows: microcytic anemia (MCV <80 fL), normocytic anemia (MCV 80-95 fL), and macrocytic anemia (MCV >95 fL) [1].

In 2019, over half a billion women aged 15-49 globally encountered 29.9% anemia, with non-pregnant women at 29.6% and pregnant women slightly higher at 36.5%. Children aged 6-59 months experienced 39.8% global anemia prevalence, with the African Region reporting the highest at 60.2% [2]. This study finds the 2019 global anemia burden, with 1.8 billion affected individuals, causing 50.3 million Years Lived with Disability. Children under 10 face the highest burden, mainly due to iron deficiency impacting psychomotor development. Gender differences reveal higher anemia rates in young females in the reproductive age group, influenced by factors like menstrual bleeding and pregnancy-related issues, despite a slight prevalence decrease [3]. In Bangladesh, where rates of iron deficiency anemia (IDA) in children exceed 55%, especially in rural areas, conventional supplementation faces challenges. This study explores a sustainable alternative using iron-fortified lentils, a staple food [4]. The prevalence of anemia in a healthy population of children (18 months to seven years) and women (14 to 30 years) during 2006-2007 was assessed in Rio Grande do Sul, Brazil. Anemia remained prevalent across different socioeconomic classes, including lower and upper classes [5].

Adolescent children (11 to 18 years) benefited from various iron supplementation types, non-pregnant women (19 to 49 years), and pregnant women (15 to 49 years) improved with iron therapy [6]. The author underscores the positive impact of intermittent iron supplementation, alone or with other micronutrients, in reducing anemia and enhancing iron stores in menstruating women, supported by consistent hematological responses in studies providing 60 mg or more of elemental iron weekly [7].

This study aims to determine the prevalence of IDA among teenage girls aged 13 to 19 years attending outpatient departments (OPDs). This age group is particularly important because menstruation increases the risk of iron deficiency, yet this issue is often overlooked in this population. The goal of this study is to identify this problem and highlight the need for targeted dietary counseling for adolescents to address iron deficiency and improve their overall health.

## Materials and methods

Study design

This cross-sectional study was conducted at Fatima Memorial Hospital, Lahore, Pakistan, from April 2024 to November 2024, involving 165 participants.

Population

Participants were selected through a convenient sampling. The targeted population (teenage girls aged 13 to 19 years) focused on those with moderate anemia (Hb between 8 and 10 g/dL) and severe anemia (Hb below 8 g/dL), as mild anemia (Hb between 10 and 12 g/dL) is sometimes temporary and may resolve without intervention. Exclusion criteria included known cases of hemolysis (Hereditary Spherocytosis, sickle cell disease, thalassemia, autoimmune hemolytic anemia, or G6PD deficiency) or autoimmune disorders (SLE and vasculitis), significant organ dysfunction (hepatitis, chronic kidney disease, or chronic liver cirrhosis), recent blood loss, psychiatric illness, cognitive impairment, or ongoing treatment for anemia.

Statistical analysis

Age and categorical variables (gender, symptoms, and yeast presence) were analyzed as frequencies and percentages. IBM SPSS 26 (IBM Corp., Armonk, NY, USA) was used; categorical variables were analyzed using percentages, and continuous variables were reported as mean ± SD. A p-value of ≤0.05 was considered statistically significant, with a 95% confidence interval applied. Associations were measured using the Chi-square test. The diagnostic workup for anemia involves a complete blood count (CBC), as it is a cost-effective and essential test to evaluate overall blood health in patients; those patients who have Hb less than 10 undergo serum iron, serum ferritin, and TIBC. No intervention has been used for study purposes, though supplements were provided for the treatment of anemia.

Ethical consideration

Ethical approval for the study was obtained from the Institutional Review Board (IRB) of Fatima Memorial Hospital, Lahore, Pakistan (FMH-10/1/2024-IRB 1356). Informed consent was obtained from all participants; individuals aged 16 or older provided consent themselves, while for participants under 16, consent was provided by their parents or guardians. Confidentiality and anonymity were maintained throughout the research process.

## Results

**Figure 1:**
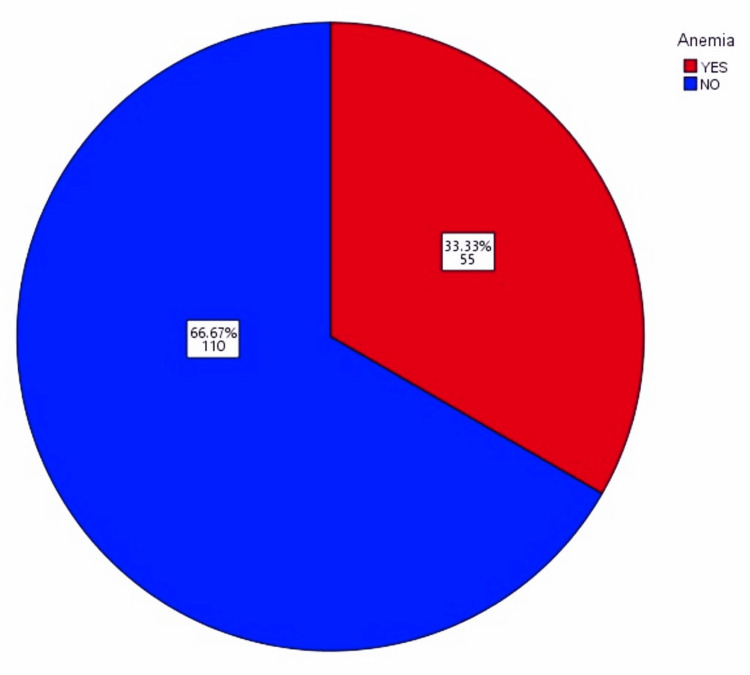
Percentage distribution of anemia among teenage girls This pie chart illustrates the distribution of anemia severity among participants. Approximately 33.33% of participants have moderate anemia (Hb 8-10 g/dL) or severe anemia (Hb <8 g/dL), while 66.67% either have no anemia or mild anemia (Hb >10 g/dL).

This study revealed that 55 (33.33%) participants were anemic, while 110 (66.67%) were non-anemic (Figure 1). Participants were divided into two main groups: anemic and non-anemic. Anemic participants were further classified into those with moderate anemia and severe anemia. The non-anemic group included individuals with mild anemia as well as those with Hb levels above 12 g/dL. Since mild anemia usually resolved without intervention, participants with Hb levels above 10 g/dL were categorized as non-anemic.

The majority, 135 (81.8%) individuals, were unaware of these preventative measures, revealing a significant knowledge gap regarding anemia prevention. Among the participants, 20 (12.1%) had Hb levels below 8 g/dL, while 35 (21.2%) had levels between 8 g/dL and 10 g/dL. The majority, 50 (30.3%) had Hb levels between 10 g/dL and 12 g/dL, and 60 (36.4%) had levels above 12 g/dL (Table 1).

**Table 1:** Percentage distribution of anemia classification Hb: hemoglobin

Sr. no.	Anemia classification (mg/dL)	Frequency (%)
1	Hb 10-12	50 (30.3)
2	Hb 8-10	35 (21.2)
3	Hb ≤8	20 (12.1)
4	Hb ≥12	60 (36.4)

The sample consisted of 165 individuals aged 14 to 19 years, with a mean age of 17.27±1.492 years. The age of menarche ranged from nine to 16 years, with a mean of 12.5±2.53 years and the most common age reported was 13. Additionally, 160 (97.0%) individuals had started menstruating, while five (3.0%) had not. Only 30 (18.2%) participants reported knowledge of anemia prevention methods, such as consuming iron-rich and vitamin C-rich foods and being aware of factors that affect iron absorption. Among the participants, 135 (81.8%) resided in urban areas, while 30 (18.2%) lived in rural areas. Five (3.0%) were illiterate, 50 (30.3%) had completed matriculation, 65 (39.4%) had attained an intermediate or equivalent level and 45 (27.3%) had education less than high school. Regarding household income, 75 (45.5%) report earnings under $178, 45 (27.3%) between $178 and $356, and another 45 (27.3%) over $356 (Table 2).

**Table 2:** Demographic profile

Sr. no.	Variables	Subcategory	Frequency (%)
1	Residential area	Urban areas	135 (81.8%)
		Rural areas	30 (18.2%)
2	Education	No education	5(3.0%)
		Matric/O-levels	50 (30.3%)
		Inter/A-levels	65 (39.4%)
		Less than high school	45 (27.3%)
3	Income	Less than 50000	75 (45.5%)
		Between 50000 and 100000	45 (27.3%)
		More than 100000	45 (27.3%)
4	Knowledge of anemia prevention methods	Yes	30 (18.2%)
		No	135 (81.8%)

Hb levels range from 6.60 g/dL to 14.00 g/dL, with an average of 10.75±2.23 g/dL. MCV varies from 66.80 fL to 100.00 fL, with an average of 80.63±8.57 fL. Serum ferritin levels span from 5.50 ng/mL to 147.00 ng/mL, with an average of 68.94±51.19 ng/mL. Serum total iron-binding capacity (TIBC) ranges from 181.00 µg/dL to 482.00 µg/dL, with a mean of 312.06±65.83 µg/dL, while serum iron levels range from 11.00 µg/dL to 140.00 µg/dL, averaging 73.33±44.46 µg/dL (Table 3).

**Table 3:** Blood indices TIBC: total iron-binding capacity; Hb: hemoglobin

Sr. no.	Variables	Mean±SD
1	Hb	10.75±2.23 g/dL
2	MCV	80.63±8.57 fL
3	Serum ferritin	68.94±51.19 ng/mL
4	Serum TIBC	312.06±65.83 µg/dL
5	serum iron levels	73.33±44.46 µg/dL

In terms of MCV, 15 (9.1%) have levels between 60 and 68 femtoliters, 45 (27.3%) fall within the 69 to 76 femtoliters range, and the majority, 105 (63.6%), have MCV levels above 76 femtoliters. Among participants with anemia, 50 (30.3%) have menstrual cycles lasting 21-35 days, five (3.0%) have cycles longer than 35 days, and none have cycles lasting four days, while among those without anemia, 100 (60.6%) have cycles lasting 21-35 days, five (3.0%) have longer cycles and five (3.0%) have cycles lasting four days, with Chi-square tests showing no significant association (χ²=3.750, p>0.05). However, 30 (18.2%) individuals with anemia experience heavy or irregular bleeding compared to 40 (24.2%) without anemia, with a significant association between anemia and heavy or irregular menstrual bleeding (χ²=4.962, p<0.05). Regarding dietary habits, 20 (12.1%) individuals with anemia follow a mostly vegetarian diet, five (3.0%) are mostly non-vegetarian, and 30 (18.2%) have a balanced diet, compared to 30 (18.2%) individuals without anemia who are mostly vegetarian, none who are mostly non-vegetarian, and 80 (48.5%) who have a balanced diet. A significant association was observed between anemia and dietary habits (χ²=12.818, p<0.05). Additionally, 15 (9.1%) individuals with anemia take dietary supplements, mostly folic acid, compared to 150 (90.9%) who do not, showing a significant association between anemia and supplement use (χ²=8.250, p<0.05). Lastly, 50 (30.3%) participants with anemia have recently experienced symptoms of fatigue, weakness, or dizziness, compared to 70 (42.4%) individuals without anemia reporting these symptoms, with Chi-square tests indicating a significant association between anemia and these symptoms (χ²=13.750, p<0.001) (Table 4).

**Table 4:** Anemia and its association with different variables

Sr. no.	Variables	Groups	Category	Frequency (%)	χ²	P-Value
1	Duration of the menstrual cycle	Anemic (N=55)	Menstrual cycles less than 21 days	0 (0.0%)	3.750	0.153
			Menstrual cycles lasting 21-35 days	50 (30.3%)		
			Menstrual cycles longer than 35 days	5 (3.0%)		
		Non-anemic (N=110)	Menstrual cycles less than 21 days	5 (3.0%)		
			Menstrual cycles lasting 21-35 days	100 (60.6%)		
			Menstrual cycles longer than 35 days	5 (3.0%)		
2	Heavy or irregular menstrual bleeding	Anemic (N=55)	Yes	30 (18.2%)	4.962	0.026
			No	25 (15.2%)		
		Non-Anemic (N=110)	Yes	40 (24.2%)		
			No	70 (42.4%)		
3	Dietary habits	Anemic (N=55)	Vegetarian diet	20 (12.1%)	12.818	0.002
			Non-vegetarian	5 (3.0%)		
			Balanced diet	30 (18.2%)		
		Non-anemic (N=110)	Vegetarian diet	30 (18.2%)		
			Non-vegetarian	0 (0%)		
			Balanced diet	80 (48.5%)		
4	Dietary supplements	-	Yes	15 (9.1%)	8.250	0.004
		-	No	150 (90.9%)		
5	Symptoms of anemia (fatigue, weakness, or dizziness)	Anemic (N=55)	Yes	50 (30.3%)	13.750	0.000
			No	5 (3.0%)		
		Non-anemic (N=110)	Yes	70 (42.4%)		
			No	40 (24.2%)		

**Figure 2:**
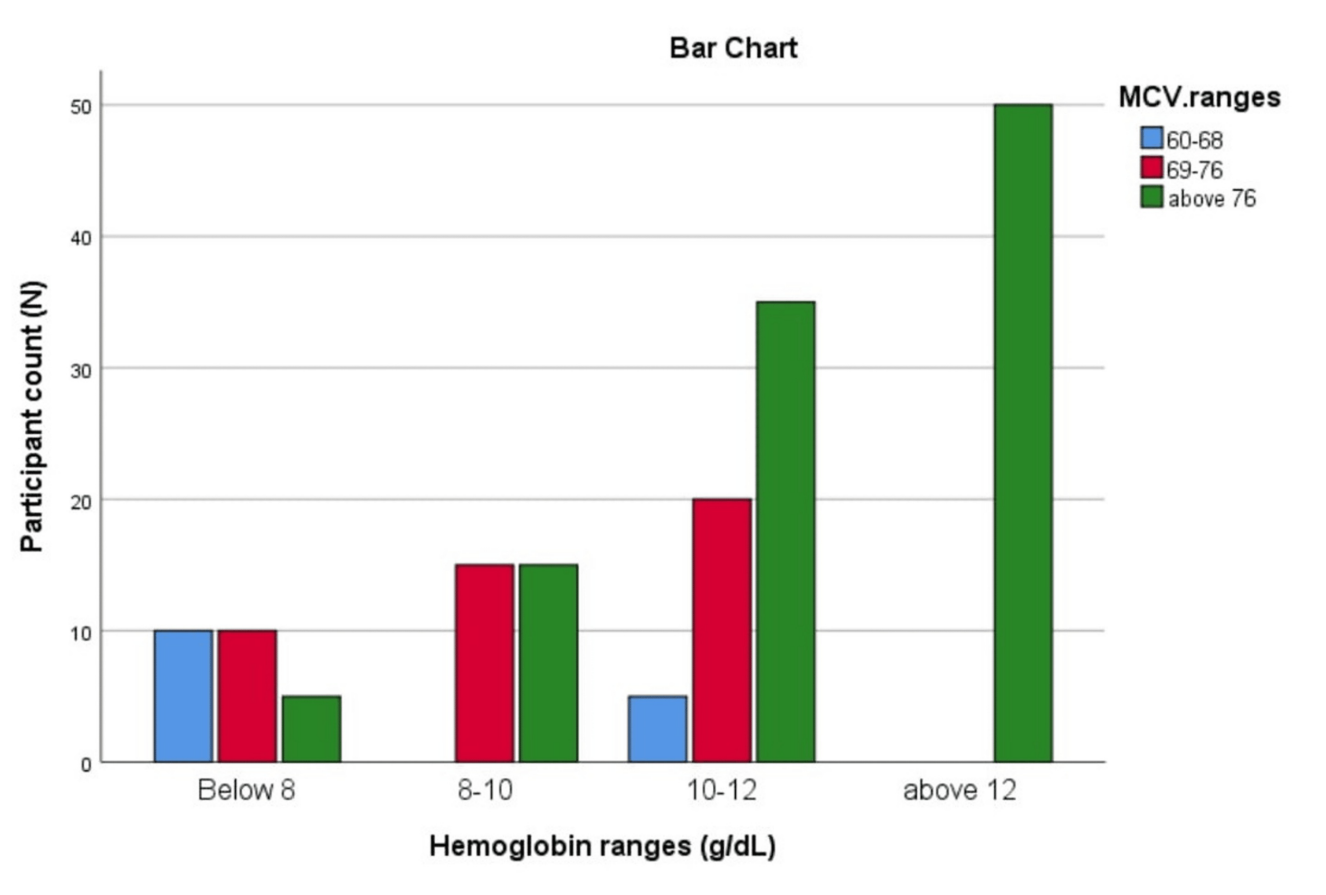
Distribution of Hb ranges by MCV ranges The bar chart indicates a rising trend in both MCV and participant count with higher Hb levels. Hb: hemoglobin; MCV: mean corpuscular volume

The crosstabulation of Hb and MCV shows clear patterns. Among individuals with Hb below 8, 10 (6.1%) have MCV in both the 60-68 and 69-76 ranges, and five (3.0%) have MCV above 76, totaling 25 (15.2%). In the 8-10 Hb range, 15 (9.1%) fall into both the 69-76 and above 76 categories, while those with Hb below 8 are zero, making up 30 (18.2%). For Hb levels between 10-12, five (3.0%) have MCV in the 60-68 range, 20 (12.1%) in the 69-76 range, and 35 (21.2%) above 76, totaling 60 (36.4%). Those with Hb above 12 show no cases in the 60-68 or 69-76 ranges, but 50 (30.3%) are above 76. A Chi-Square test (χ²=74.206, p<0.001) confirms a statistically significant association between Hb levels and MCV ranges. The bar chart indicates a rising trend in both MCV and participant count with higher HB levels (Figure 2).

## Discussion

This study analyzed anemia prevalence and associated factors in a cohort of 165 individuals, with 55 (33.33%) participants having moderate or severe anemia and 110 (66.67%) having no or mild anemia. Only 30 (18.2%) reported knowledge of anemia prevention, highlighting a significant awareness gap. Anemia was significantly associated with dietary habits, heavy or irregular menstrual bleeding, and dietary supplements. Participants with anemia were more likely to report symptoms such as fatigue, weakness, and dizziness. The observed trend of higher HB levels correlating with higher MCV suggests that traits like thalassemia, typically associated with lower MCV, can be excluded in this population.

Hypo-proliferative anemias are divided into microcytic (MCV <80 fL), normocytic (MCV 80-100 fL), and macrocytic (MCV >100 fL) varieties. It was determined that all of the patients in our targeted population with moderate anemia (Hb <10g/dL) had an MCV of less than 80 fL, indicating that they were all classified as having microcytic anemia. One-fifth of adolescent girls had anemia, with approximately half of these cases being chronic and primarily due to IDA. According to WHO criteria, 19.9% of teenage girls had anemia, with iron deficiency and anemia being seven and eleven times more common in girls compared to boys, respectively. Thirty percent of teenagers and women experiencing heavy menstrual bleeding (HMB) have serum ferritin levels less than 15 ng/mL. While mild-to-moderate IDA frequently shows no symptoms at all, when it does, the most common ones are exhaustion, weakness, and dyspnea. Headaches, pica, hair loss, brittle nails, cold intolerance, and restless leg syndrome are some additional possible symptoms. Similar findings were observed in our study, indicating a strong correlation between anemia and these symptoms [8]. Anemia is a symptom of an underlying condition rather than a disease itself. It is frequently categorized according to its biological processes [9,10].

Young women are disadvantaged due to physiological iron losses from menstruation and increased iron demands. Access to medical care is crucial in preventing excessive blood loss during menarche. Our study emphasizes that teenage girls often lack proper counseling on dietary modifications during menarche, especially if they face socioeconomic constraints [11-13]. Nutritional deficiencies play a key role in the development of anemia. Essential nutrients such as iron, cobalt, magnesium, and micronutrients including vitamins A, folate, B6, B12, and other B vitamins, are critical for RBC formation, Hb synthesis, iron absorption, and overall cellular function. Our findings reveal a significant association between dietary habits and anemia. It suggests that dietary habits, particularly lacking essential nutrients, may contribute to the higher prevalence of anemia in specific populations [14,15]. Vitamin A deficiency exacerbates IDA by affecting (1) iron release from liver stores, (2) enhanced iron absorption, (3) iron accumulation in macrophages, and (4) suppressed erythropoiesis, resulting in anemia [16]. Developing reliable biomarkers for iron status remains challenging, as iron deficiency is marked by reduced Hb and ferritin-bound iron levels, increased TIBC, and low transferrin saturation. Ferritin, an indirect marker of total iron, can rise during inflammation, infections, or cancer-related anemia, one of our exclusion criteria [17-19].

Globally, 1.62 billion people are affected by anemia, with an estimated 36% of the population in developing nations being impacted. Anemia significantly affects 305 million school children (25.4%). In Sri Lanka, 47.8% of female children aged 12-18 years are reported to have iron deficiency. In Nepal, 65.6% of adolescents aged 10-19 years are anemic, while in rural Kazakhstan, the prevalence among school children is reported to be 13%. In Arab countries of the Persian Gulf, the prevalence ranges from 12.6% to 50%, and Turkey reports a prevalence of 12.5%. The Philippines sees anemia affecting nearly 70% of infants, 30% of preschool children, 40% of school children, and pregnant women. Prevalence rates for anemia among school children in Mexico, Colombia, and the United States are 11.6%, 4.2%, and 3.6%, respectively. Our study shows that 33.33% of participants have moderate anemia (Hb 8-10 g/dL), while 66.67% either have no anemia or mild anemia (Hb >10 g/dL) [20]. Iron deficiency in adolescent females often causes fatigue and cold intolerance, which are relieved by oral iron therapy. Despite a global decline in anemia, high prevalence persists in certain US groups and developing countries, where nearly 50% of young children are affected. In developed countries, IDA is common in adolescent females with HMB. Our study found no link between menstrual cycle length and anemia, but a significant association with irregular menstrual bleeding. Around 30% of females with HMB have iron deficiency, and iron therapy improves symptoms like fatigue and weakness, as confirmed by our study [21,22].

Oral iron therapy is a treatment option for isolated iron deficiency. To address this, microencapsulated iron supplements known as "Sprinkles" are used as both fortification and supplementation, with programs implemented or planned in countries such as Mongolia, Indonesia, Pakistan, Bangladesh, Bolivia, and Haiti [23]. Socioeconomic status significantly impacts IDA prevalence, with higher rates observed in rural areas compared to urban settings, according to the NFHS IV and V surveys. In our study, 135 participants (81.8%) reside in urban areas, while 30 (18.2%) are from rural areas. A notable number of individuals in both settings lack awareness about anemia; only 30 participants (18.2%) reported knowledge of prevention methods, such as consuming iron and folic acid, while 135 individuals (81.8%) were unaware of these measures, highlighting a significant knowledge gap regarding anemia prevention [24].

On October 2, 1975, the Integrated Child Development Services (ICDS) program was launched, becoming one of India's most significant initiatives for early childhood development. The four primary components of this program are Early Childhood Care, Education, and Development (ECCED), Health Services, Community Mobilization, Awareness, Advocacy, Information, Education, and Communication, Care and Nutrition Counseling. Similar initiatives, like seminars, door-to-door awareness campaigns, or symposiums, should be implemented to inform communities about the prevention of anemia. To close the substantial knowledge gap, these programs could concentrate on dietary recommendations, iron supplementation, and general health awareness, particularly in rural and urban populations [25].

Study limitations

The study utilized a convenience sampling method, which may not accurately represent the region's broader population of teenage girls. A larger, randomized sample would strengthen the findings. The absence of follow-up data limits understanding of how anemia may change over time in this population. Reliance on self-reported dietary habits and menstrual health can introduce bias, as participants may underreport or misreport their experiences. The study was conducted in a single hospital in Lahore, limiting its generalizability. Resource constraints and non-response bias could further affect data completeness. 

## Conclusions

This study shows a high prevalence of IDA in teenage girls, particularly around menarche when iron needs increase. Its impact on physical growth, cognitive development, and future maternal health is of critical importance. The strong association between dietary habits, menstrual irregularities, and anemia highlights the multifaceted nature of this health issue. Addressing these factors through community education and targeted interventions can enhance the understanding and management of anemia in this vulnerable population, ultimately leading to improved health outcomes.
